# Four-tract tractography: multiparametric direct targeting of the dentatorubrothalamic tract

**DOI:** 10.3171/2024.7.FOCVID2483

**Published:** 2024-10-01

**Authors:** Andreas Seas, Katrina Hon, David Chung, Lynne Todd, Bhavya R. Shah, Shivanand P. Lad, Stephen Harward

**Affiliations:** 1 Department of Neurosurgery, Duke University School of Medicine, Durham, North Carolina;; 2 Department of Biomedical Engineering, Duke Pratt School of Engineering, Durham, North Carolina;; 3 Department of Radiology, Transcranial Focused Ultrasound Laboratory and Program, UTSW Medical Center, Dallas, Texas;; Departments of 4 Radiology, Neuroradiology and Neuro-intervention Section and; 5 Neurological Surgery, UTSW Medical Center, Dallas, Texas

**Keywords:** focused ultrasound, essential tremor, ablation, thalamotomy, tractography

## Abstract

This video article presents a case study of a 70-year-old male with medically refractory essential tremor treated with magnetic resonance–guided focused ultrasound (MRgFUS). Following an initial successful ablation of the right thalamus, the patient underwent left-sided thalamotomy. After two tractography-guided sonications, the authors observed a significant reduction in his right-hand tremor with no immediate side effects. Postprocedure evaluation revealed sustained tremor reduction with minimal side effects, showcasing bilateral MRgFUS as an effective, noninvasive option for essential tremor management.

The video can be found here: https://stream.cadmore.media/r10.3171/2024.7.FOCVID2483

## Transcript

In this video article, we will examine a case of medically refractory essential tremor treated using four-tract tractography: multiparametric direct targeting of the dentatorubrothalamic tract (DRTT).^1,2^

### 0:33 Description of Four-Tract Tractography.

Four-tract tractography is a precision medicine, multiparametric, advanced imaging approach developed by Dr. Bhavya Shah at UT Southwestern that uses patient-specific imaging to identify the treatment target for MRgFUS. It centers around targeting the confluence of the decussating and nondecussating dentatorubrothalamic tracts, while avoiding the adjacent medial lemniscus and corticospinal tracts.^3,4^

### 0:58 Patient History.

We present the case of a 70-year-old left-handed male who first noted a left-hand tremor at the age of 15. Over time, his tremors progressed to involve both hands. Due to their severity, he noted marked interference with his work and activities. He had tried adequate doses of diazepam, propranolol, and primidone without benefit.

### 1:20 Initial Examination and Treatment.

On initial examination, he had no hand tremor at rest, just with posture and action, consistent with a diagnosis of essential tremor. Nine months earlier, he had undergone successful right-sided MRgFUS thalamotomy, targeting the confluence of the decussating and nondecussating dentatorubrothalamic tracts, with a self-reported 80% improvement in tremor without significant side effects. As such, he presented to our clinic for consideration of a second-sided MRgFUS treatment for his remaining right-hand tremor.^5,6^

In this video, we show the patient undergoing an MR-guided focused ultrasound lesion of the left thalamus. The choice of thalamotomy over other neurosurgical options was governed by the patient’s desire to undergo a treatment that would not require an implanted device but would lead to rapid onset of benefit.

### 2:07 Patient Consent.

Note that our patient consented to the collection and sharing of his medical data and the upcoming photos and videos for research publication

### 2:15 Baseline Assessment.

In preparation for this procedure, we completed a baseline tremor assessment using the standard Clinical Rating Scale for Tremor (CRST).^7^ Additionally, we completed a Scale for the Assessment and Rating of Ataxia (SARA) in order to assess baseline gait and balance as imbalance following MRgFUS is a well-documented side effect.^1,8^

### 2:33 Calculation of Skull Density Ratio.

We obtained a CT scan to assess the patient’s skull density ratio (SDR), a critical factor in predicting the ability of ultrasound to safely and effectively penetrate the skull to create a lesion.^2^ A value of 0.40 or higher is considered appropriate per Medicare guidelines. This patient’s SDR was 0.54 as per his initial CT scan evaluation.

### 2:57 Preoperative Tractography-Based Targeting.

To identify the appropriate anatomical targeting for this patient, we utilized advanced MR imaging along with the previously described four-tract tractography approach.^3,4^ Specifically, we started with consensus coordinates to define a preliminary target (14 mm lateral of AC-PC, 25% anterior of PC, and 1.5 mm superior of AC-PC). The AC-PC distance in this patient was 26.1 mm, so the consensus coordinates were 14 mm lateral, 6.5 mm anterior, and 1.5 mm superior of AC-PC. We then identified the corticospinal tract (CST), medial lemniscus (ML), and both the decussating and nondecussating dentatorubrothalamic tracts (DRTTs) and overlaid them on FGATIR images. We adjusted our target so that our predicted lesion would be just medial of CST and covering the DRTT—an anatomical region that often yields excellent tremor control.^9^ We then refined our target using tractography performed by the commercially available Brainlab software. We settled on an initial target of 13 mm lateral, 5.5 mm anterior, and 1.2 mm superior of AC-PC. Notably, these target coordinates are medial, posterior, and inferior to the target predicted by consensus coordinates alone, thus highlighting how a multiparametric approach can afford greater targeting precision for improving efficacy and reducing side effects.

### 4:19 Head Shave and Frame Placement.

On the day of the procedure, our patient underwent a complete head shave followed by injection of local anesthetic and placement of a head frame. Note how the head frame is positioned slightly lower than is typical for deep brain stimulation procedures (along the orbital ridge and just below the inion).

### 4:35 Patient Transfer, Membrane Placement, and Positioning.

The patient is transferred to the MRI suite where the membrane is placed over the scalp and the attached frame. We take care to ensure the membrane is low and flush along the scalp, minimizing folds as much as possible. The patient is then laid on the MRI table, the frame is attached to the table, and the membrane is connected to the ultrasound transducer. The membrane is then filled with cold water. Care is taken to ensure that air bubbles are minimized.

### 5:00 Initial Imaging and Identification of Anterior and Posterior Commissures.

The patient is put into the MRI and localizing T1 images are obtained. Once completed, the anterior and posterior commissures are selected on the localizing images and the predetermined target from our planning is selected. The ultrasound is then adjusted in all axes to ensure the natural focus of the ultrasound overlies our intended target.

### 5:19 CT Scan Merge and Exclusion of Membrane Folds.

Once aligned, the CT scan used for calculating the skull density before the procedure is uploaded and fused to the localizing images. Areas that we do not want the ultrasound to traverse (such as membrane folds) are marked off.

### 5:33 Alignment Sonications.

Three alignment sonications are done to ensure tissue heating at our target is properly positioned in the anterior-posterior, right-left, and superior-inferior directions. During these alignments, the target tissue temperature is between 42°C and 45°C.

### 5:47 First Verification Sonication and Patient Assessment.

Once the ultrasound and its associated tissue heating are aligned, a verification sonication is done with a goal of achieving a target tissue temperature of 48°C–52°C—not enough to permanently damage the tissue but enough to have a temporary stunning effect. At this point, the patient is reexamined in the MRI suite for side effects (specifically numbness and tingling, motor weakness, incoordination/ataxia, taste changes, or speech difficulties) as well as potential tremor benefit.

For this patient, our first verification sonication achieved a peak temperature of 51°C. However, we saw only minimal tremor benefit.

### 6:25 Target Adjustment.

We thus returned to our tractography data. We saw that we still had some room to move posteriorly toward the medial lemniscus, which could explain the lack of tremor response. As such, we adjusted our target to 13 mm lateral, 5 mm anterior, and 1.2 mm superior.

### 6:41 Second Verification Sonication and Patient Assessment.

We performed a second verification sonication where we once again reached a peak temperature of 51°C. This time, the tremor benefit was much more obvious without any evidence of side effects.

### 6:51 First Treatment Sonication and Patient Assessment.

As such, we then proceeded to treatment sonications, where our goal is to reach 57°C for 1 second to induce permanent tissue damage.^10^ As heating develops, we are monitoring the rate of heating as well as looking for signs of cavitation. For this patient, the first treatment sonication reached a temperature of 54°C. While it did not reach our intended goal of 57°C, it did sustain at 54°C for quite some time and thus likely induced a small lesion.

Following this first treatment, we reexamined the patient, confirming good tremor benefit without side effect.

### 7:23 Second Treatment Sonication and Patient Assessment.

To further improve our tremor benefit, we moved our target superior, lateral, and anterior (13.5 lateral, 5.5 anterior, and 2.5 superior of the AC-PC line and PC point). This roughly follows the course of the DRTT tracts as they course up to join the CST. Note that this sonication of a second site is not universally accepted. For this treatment, we reached 56°C and held it for several seconds.

Following this treatment, the patient’s tremor was largely gone, and importantly, he had no obvious side effects.

### 7:53 Head Frame Removal.

Satisfied with the results, we removed the patient from the MRI and took off his head frame.

### 7:58 Postprocedure MRI.

We obtained a postprocedure MRI to confirm an adequate lesion and then transferred the patient to recovery.

### 8:02 Immediate Postoperative Results.

After approximately 45 minutes in recovery, the patient was up and walking, taking good oral intake, and using the restroom independently. He was subsequently discharged home.

### 8:14 One-Week Follow-Up Visit.

He returned to clinic 1 week later for a follow-up tremor evaluation. At that time, his CRST score (parts A and B) for his right hand improved from 22.5 to 4, an 82% decrease. Importantly, in terms of side effects, he noted only mild gait and balance abnormalities during our clinic evaluation that fully resolved by 3 weeks postprocedure.

## Acknowledgments

Thank you to the outstanding Duke MRI team for their help supporting this effort.

## Disclosures

Dr. Shah reported consulting fees from Insightec outside the submitted work. Dr. Harward reported consulting fees from Insightec during the conduct of the study.

## Author Contributions

Primary surgeon: Harward. Editing and drafting the video and abstract: Seas, Hon, Chung, Shah, Harward. Critically revising the work: Seas, Hon, Todd, Shah, Lad, Harward. Reviewed submitted version of the work: all authors. Approved the final version of the work on behalf of all authors: Seas. Supervision: Shah, Lad, Harward. Developed 4TT: Shah.

## Supplemental Information

### Patient Informed Consent

The necessary patient informed consent was obtained in this study.
